# Supplementary figures and images for: Evolutionary analysis of the OSCA gene family in sunflower (Helianthus annuus L) and expression analysis under NaCl stress
Source: PeerJ. 2023 Apr 17;11:e15089. doi: 10.7717/peerj.15089 (PMC10117387; doi:10.7717/peerj.15089)

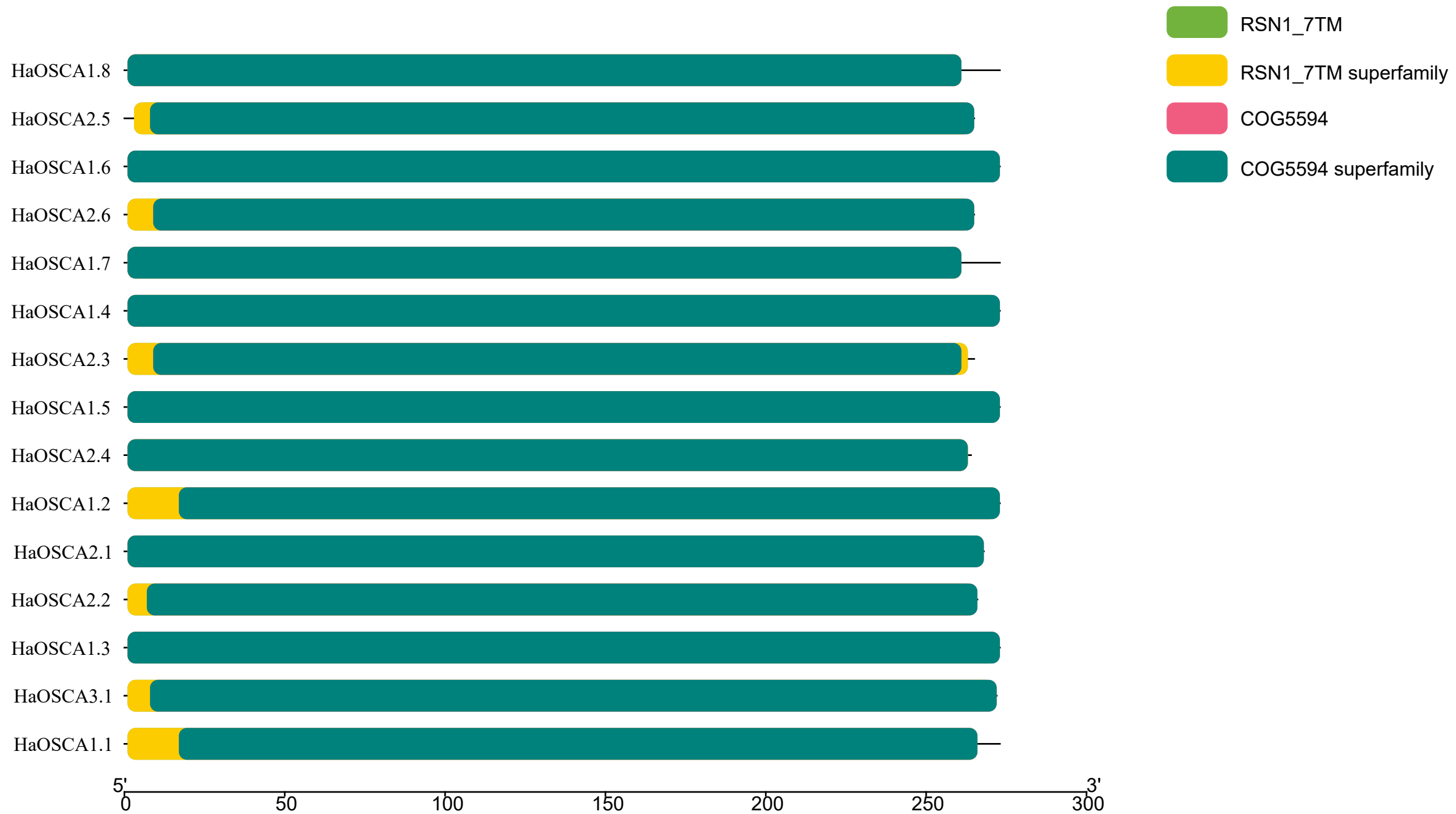

Supplement: Figure S1 [file peerj-11-15089-s001.pdf]
